# Swing Trend Prediction of Main Guide Bearing in Hydropower Units Based on MFS-DCGNN

**DOI:** 10.3390/s24113551

**Published:** 2024-05-31

**Authors:** Xu Li, Zhuofei Xu, Pengcheng Guo

**Affiliations:** School of Water Resources and Hydroelectric Engineering, Xi’an University of Technology, Xi’an 710048, Chinaguoyicheng@xaut.edu.cn (P.G.)

**Keywords:** hydropower units, swing trend prediction, feature selection, MFS-DCGNN algorithm

## Abstract

Hydropower units are the core equipment of hydropower stations, and research on the fault prediction and health management of these units can help improve their safety, stability, and the level of reliable operation and can effectively reduce costs. Therefore, it is necessary to predict the swing trend of these units. Firstly, this study considers the influence of various factors, such as electrical, mechanical, and hydraulic swing factors, on the swing signal of the main guide bearing *y*-axis. Before swing trend prediction, the multi-index feature selection algorithm is used to obtain suitable state variables, and the low-dimensional effective feature subset is obtained using the Pearson correlation coefficient and distance correlation coefficient algorithms. Secondly, the dilated convolution graph neural network (DCGNN) algorithm, with a dilated convolution graph, is used to predict the swing trend of the main guide bearing. Existing GNN methods rely heavily on predefined graph structures for prediction. The DCGNN algorithm can solve the problem of spatial dependence between variables without defining the graph structure and provides the adjacency matrix of the graph learning layer simulation, avoiding the over-smoothing problem often seen in graph convolutional networks; furthermore, it effectively improves the prediction accuracy. The experimental results showed that, compared with the RNN-GRU, LSTNet, and TAP-LSTM algorithms, the MAEs of the DCGNN algorithm decreased by 6.05%, 6.32%, and 3.04%; the RMSEs decreased by 9.21%, 9.01%, and 2.83%; and the CORR values increased by 0.63%, 1.05%, and 0.37%, respectively. Thus, the prediction accuracy was effectively improved.

## 1. Introduction

In actual operation, the swing trend of the main guide bearings in hydropower units usually contains abundant status and fault information. Therefore, research on predicting the swing trend of the main guide bearings in hydropower units can effectively reflect the true operating status of the units and provide early warning. Due to the complex changes in the operating conditions of the hydropower units, the abnormal swing is easily affected by various factors. Therefore, when building a swing trend prediction model, it is necessary to perform multi-index fusion selection to utilize the advantages of the information and to improve the prediction accuracy. The main factors affecting the main guide bearing include the water head (H), active power (P), temperature in the oil groove of the guide bearing (T), exciting current (I), exciting voltage (U), axial displacement (D), rotation speed (S), pressure pulsations at the inlet between the guide vanes and the runner (V1), and pressure pulsation between the runner and the head cover (V2).

The appropriate features of the swing signal of the main guide bearings can improve the accuracy of fault diagnosis and prediction. However, redundant and invalid features can lead to increased computational costs and lower prediction accuracy. Therefore, through feature selection and fusion, selecting the features having a high correlation with the target signal can improve the prediction accuracy of the hydropower units. Due to the non-stationarity and strong background noise of the swing signal, the prediction algorithm has low accuracy. Therefore, determining how to eliminate the interference of noise from the massive original monitoring signals and excavate the signals reflecting the real operation state is the key focus of trend prediction. On the other hand, in the operating processes of the unit, different environmental impacts should be considered, and appropriate state variables should be selected as the reference input to improve the accuracy of the prediction algorithm.

At present, the commonly used algorithms for the swing trend prediction of the main guide bearings in hydropower units include [1] the autoregression algorithm, neural networks, support vector regression, and so on. The autoregression algorithm is mainly used to solve the linear problem, and there are certain defects in the prediction of a non-stationary swing signal [2]. Support vector regression is based on the minimization of structural components and the analysis of high-dimensional non-linear prediction problems through transformation kernels. This method has good linear fitting ability and generalization, but it is prone to overfitting problems [3,4].

The existing prediction methods fail to effectively consider the potential relationships between variables. On the one hand, the statistical vector auto-regressive algorithm (VAR) [5] and the Gaussian processes algorithm (GP) [6] are unable to explore non-linear relationships between variables. On the other hand, the LSTNet [7,8] and TPA-LSTM [9] methods, despite the fact that they can mine non-linear relationships, cannot explicitly determine the dependencies between any two variables. In the problem of time-series prediction, the method based on the graph neural network (GNN) [10] relies on a predefined graph structure and can obtain the relationships between variables. However, the given graph structure may not necessarily be optimal, and it should be continuously updated during the training process of graph learning.

At present, the status and trend prediction of hydropower units in intelligent hydropower stations still faces huge challenges. The specific problems are as follows: the accuracy and the generalization ability of the prediction models of hydropower units need to be improved. Due to the increasing complexity of hydropower units and their operating conditions, the current state evaluation and the prediction theories regarding hydropower units have bottlenecks relating to the interpretation of complex data and the description of state evolution laws. In addition, understanding how to establish a certain highly generalized and adaptive prediction model under different operating conditions also poses great challenges. The existing prediction models, such as shallow network models, support vector machines, and related vector machines, have problems such as difficulties related to autonomous learning and insufficient intelligent reasoning ability.

The contributions of this study are summarized below:

(1) The influences of various factors (e.g., electrical and mechanical factors, hydraulic swing, and so on) on the swing trend of the main guide bearing are considered. The multi-index feature selection algorithm (MFS) is used to obtain the appropriate state variables, and the low-dimensional effective feature subset is obtained through the Pearson correlation coefficient and distance correlation coefficient algorithms.
(2) The dilated convolution graph neural network (DCGNN) is used to predict the swing trend of the main guide bearing. The existing GNN methods rely heavily on predefined graph structures for prediction. The DCGNN algorithm can solve the spatial dependence between variables without defining the graph structure and can provide the adjacency matrix simulated by the graph learning layer, which avoids the problem of over-smoothing that often occurs in the graph convolution network and effectively improves the prediction accuracy.

## 2. Related Work

### 2.1. Multi-Index Features Selection

An increase in redundant and irrelevant features within the monitoring signals of hydropower units will result in higher computational costs and decreased prediction accuracy. The purpose of feature selection is to obtain an optimal subset from the input variables to accurately describe the device state. The feature selection method includes two categories: the linear correlation method [11] and the non-linear correlation method [12].

The linear correlation methods of feature selection include the following: the mutual information method [13], the maximum information coefficient method [14], the Spearman correlation coefficient method [15], and the Pearson correlation coefficient method [16,17], etc.

Zhang L et al. [18] proposed a new forward search-based feature selection algorithm, which uses the theory of mutual information and interaction information to find the optimal subset associated with multi-classification labels and reduce the computational complexity. However, the mutual information method is directly used in the feature selection process, and the variables need to be discretized first, and the results are very sensitive to the discretization method. The maximum information coefficient method (MIC) [19] first finds an optimal discretization method and then converts the value of mutual information into a measurement.

Zhang M X et al. [20] proposed an NMIC-FS method, which is able to measure the correlation with categories and the redundancy between features and realize rapid feature selection in combination with a forward sequential search strategy. However, when the data density is low, or when the number of variables is small, the maximum information coefficient may fail. The Spearman correlation coefficient method is a linear correlation analysis using the rank size of two variables and does not require the distribution of the original variables. However, it belongs to the non-parametric method and has a low test efficiency. The Pearson correlation coefficient method is good at extracting the relationships between features. Wu Hongxia et al. [21] evaluated the feature correlation with the Pearson correlation coefficient and then selected the evaluated feature subset by using the artificial ant colony algorithm.

However, the Pearson correlation coefficient method only describes the degree of linear correlation between two variables and does not consider the degree of non-linear correlation between variables. The distance correlation coefficient method can solve this problem well [22]; when the distance correlation coefficient is 0, the two variables are independent. Therefore, this paper uses the Pearson correlation coefficient and distance correlation coefficient to form the fusion feature index.

### 2.2. Swing Trend Prediction of Hydropower Units

Deep learning-based methods have recently made great progress in terms of time-series prediction [23]. Deng Y M et al. [24] proposed a swing prediction method using a CNN–BiGRU combined hydropower unit algorithm and then constructed the prediction algorithm in parallel with a bidirectional gated recurrent unit (BiGRU) network. The gated recurrent unit (GRU) [25] overcomes the problem of RNN gradient vanishing, but its long-term correlation is associated with slice data rather than the whole sequence, which may lead to overfitting and a reduction in prediction accuracy [26,27].

In a hydropower unit status sequence prediction system, monitoring the fatigue and health status of the gears is essential to ensure the reliable operation of the equipment. Using data, machine learning-related algorithms can extract useful information about gear fatigue and damage. For example, train sensor data (such as swing and temperature data) are used to identify early signs of gear failure. In addition, they can be used to predict the remaining service life of the positive gear and thus avoid accidental downtime [28]. In addition, the normal operation of hydropower units is also strongly related to the working conditions of the bearings. PIResNet is a network that combines physical knowledge and data-driven methods; it is especially suitable for the fault diagnosis of rolling element bearings. PIResNet not only considers the data characteristics but also integrates an understanding of the physical processes of bearing operation, which improves the accuracy and reliability of fault diagnosis [29].

Jiang W et al. [30] first proposed the long short-term memory (LSTM) network, which reduces the difficulty involved in learning long-term correlation by learning historical information from the sensor data. Huang C G et al. proposed a prediction method based on bi-directional long short-term memory (BLSTM), which can remember both past and future input information [31]. Sun Y H et al. [32] proposed a swing prediction algorithm for hydropower units based on the adaptive noise fully integrated empirical mode decomposition method and the LSTM neural network [33]. Shih S Y et al. [34] proposed a TAP-LSTM algorithm to select the relevant time-series and to use its frequency domain information for time-series prediction, using a set of filters to extract time-invariant temporal patterns. However, none of the above methods represent the dependence between variables and cannot effectively extract the correlation between variables.

Lai G et al. [35] developed a new deep learning framework, namely, a long short-term time-series network (LSTNet). LSTNet uses a convolutional neural network (CNN) and a recursive neural network (RNN) to extract short-term local dependence patterns between variables and find long-term patterns of time-series trends. Although the LSTNet algorithm can mine the non-linear relationships, it cannot explicitly determine the dependencies between any two variables.

The advantages of GNN are reflected in its handling of relational dependence. Yu B et al. [36] first introduced GNN into the prediction and proposed a spatio-temporal graph convolutional neural network (STGCN). Wang S et al. [37] combined GRU and GNN to transform the time-series data into graph structure data; thus, GRU and GNN were used for spatio-temporal feature extraction. Liu P H et al. [38] added a fuzzy cognitive block (FCB) to the convolutional neural network to improve the stability of the algorithm in terms of time-series prediction. Li G P et al. [39] proposed dynamic graph convolutional networks to solve the problem related to the interpretability of the parameters in the prediction. However, GNN relies on an a priori definition of the graph structure, and the explicit graph structure may have missing node relationships, which leads to the learning of inaccurate node features. Furthermore, the existing GNN methods rely heavily on predefined graph structures to perform time-series prediction.

In this paper, without defining the graph structure, the problem of spatial dependence between variables is solved. The adjacency matrix simulated by the graph learning layer is given, which avoids the problem of over-smoothing that often occurs in the graph convolution network and effectively improves the prediction accuracy.

## 3. MFS-DCGNN Swing Trend Prediction Algorithm

### 3.1. MFS Algorithm

The operation process of hydropower units is complex and, in its operation, the influences of many factors need to be considered so that the original data of the swing trend have the features of a high-dimensional and complex structure. Redundant features and invalid features increase the algorithm training time and reduce the accuracy of the algorithm prediction. Therefore, the effective feature selection method can obtain low-dimensional effective feature subsets, thereby improving the prediction accuracy of the algorithm.

The MFS algorithm uses the Pearson coefficient and distance correlation coefficient to form the fusion feature index and selects the factors that are co-correlated with the swing signal. The Pearson coefficient measures the degree of correlation between two variables by covariance, while dividing by the product of the respective standard deviation for normalization. Specifically, the Pearson correlation coefficient measures the proportion of covariability to individual variability for both variables. It characterizes the closeness of linear relationships between variables and can be used to describe the similarity or correlation of variables. The formula is as follows:

(1) $$\rho (X,Y)=\frac{cov(X,Y)}{\sigma_{X}\sigma_{Y}}$$

where X, Y represent the value of state variables affecting the state sequence of the hydropower unit, such as the temperature of the guide bearing oil tank (T), axial displacement (D), guide vane, and runner inlet pressure pulse (V1), etc. ρ(X,Y) represents the Pearson correlation coefficient between the variables X and Y, cov indicates the covariance, and $\sigma_{X}$ and $\sigma_{Y}$ indicate the standard deviation of X and Y, respectively.

Overcoming the disadvantages of the traditional Pearson coefficient, the distance correlation coefficient can measure the relationship between non-linear correlation variables. In some cases, even if the Pearson correlation coefficient is 0, the two variables cannot be determined as linear irrelevant (possibly non-linear correlation), but if the distance correlation coefficient is 0, the two variables can be judged as independent $FOR\;X\in R^{p},Y\in R^{q},(X,Y)=\{(x_{i},y_{i}):i=1,2,\ldots n\}$ is the observed random sample, and the distance correlation coefficient between X and Y can be defined as follows:

(2) $$R^{2}\left(X,Y\right)=\frac{v^{2}\left(X,Y\right)}{\sqrt{v^{2}\left(X,Y\right)v^{2}\left(X,Y\right)}}$$

(3) $$v^{2}\left(X,Y\right)=\frac{1}{n^{2}}\sum_{i,j=1}^{n}A_{i,j}B_{i,j}$$

(4) $$v^{2}(X,X)=\frac{1}{n^{2}}\sum_{i,j-1}^{n}A_{i,j}^{2}$$

(5) $$v^{2}\left(Y,Y\right)=\frac{1}{n^{2}}\sum_{i,j-1}^{n}B_{i,j}^{2}$$

$A_{i,j}$ and $B_{i,j}$ can be described as follows:

(6) $$A_{i,j}={\left\|x_{i}-x_{j}\right\|}_{2}-\frac{1}{n}\sum_{k=1}^{n}{\left\|x_{k}-x_{j}\right\|}_{2}-\frac{1}{n}\sum_{l=1}^{n}{\left\|x_{i}-x_{l}\right\|}_{2}+\frac{1}{n^{2}}\sum_{k,l=1}^{n}{\left\|x_{k}-x_{l}\right\|}_{2}$$

(7) $$B_{i,j}={\left\|y_{i}-y_{j}\right\|}_{2}-\frac{1}{n}\sum_{k=1}^{n}{\left\|y_{k}-y_{j}\right\|}_{2}-\frac{1}{n}\sum_{l=1}^{n}{\left\|y_{i}-y_{l}\right\|}_{2}+\frac{1}{n^{2}}\sum_{k,l=1}^{n}{\left\|y_{k}-y_{l}\right\|}_{2}$$

The MFS algorithm analyzes the linear and non-linear relationship between two variables and proposes the fusion feature, which is formulated as follows:

(8) $$CF(X,Y)=\frac{\left|\rho (X,Y)\right|+R^{2}(X,Y)}{2}$$

### 3.2. DCGNN Swing Trend Prediction Method

#### 3.2.1. Temporal Convolution and GNN Based on Dilated Convolution

Temporal convolution (TC) is a convolution network used to process time series data. A temporal convolution network can calculate the probability of the element in the future time point t according to the order of elements in a known time series.

**Figure 1:**
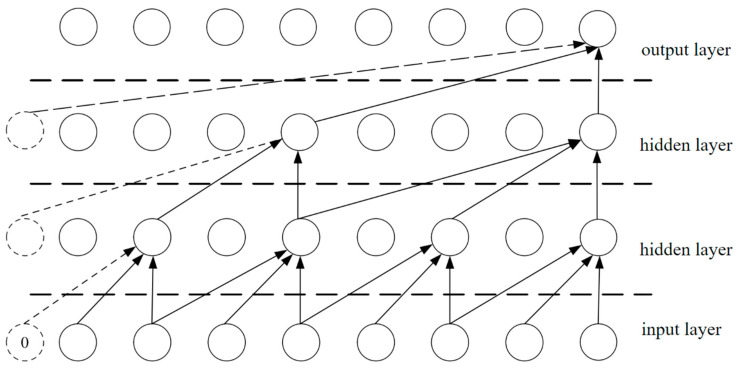
Structure diagram of the dilated convolution model with the dilated factor of 3.

In order to calculate the value of the future moment by using the larger visual field domain, it is necessary to set a larger dilated convolution to make more elements participate in the prediction and realize the growth of the visual field domain. In a temporal convolutional network, the future moment refers to the time point after the current time point, which is not yet coming but will come relative to the current time. The visual field domain refers to the size of the region that the convolution operation can see in the input data. The dilated factor refers to the interval size between the adjacent neighbors of the convolution kernel in the dilated convolution. In conventional convolution operations, the convolution kernel slides on the input data with a stride of 1, with the interval between intermediate adjacent elements of 1. However, in the dilated convolution, the receptive field of the convolution kernel can be increased, thus capturing a wider range of input information. Figure 1 shows the model diagram of the dilated convolution.

There are four layers in the figure above. The first layer is the output layer, the second layer is the hidden layer, the third layer is the hidden layer, and the fourth layer is the input layer.The dotted lines and “0” in the diagram indicate zero-padding at the beginning of the sequence, enabling convolutional operations on the nodes at the beginning of the sequence.

Dilated convolution uses a one-dimensional full convolutional network, where the length of each hidden layer is the same as the length of the input layer, and padding length is added to maintain the same length as the subsequent layers before. The sequence $X=\left\{x_{1},x_{2},\ldots ,x_{r}\right\}$ for the dilated convolution calculation formula at $x_{t}$, is shown in Formula (9).

(9) $$F\cdot_{d}X(x_{t})=\sum_{k=1}^{K}f_{k}x_{t-(K-k)d}$$

where ⋅ represents the convolution operation, d is the dilated factor, and the hidden layer is $F=\{f_{1},f_{2},\ldots f_{k}\}$.

GNNS is a graph composed of a given point and a straight line connecting any two points. It is usually used to describe a specific relationship between the transactions represented by these points. In a graph, the state of a node depends on the state of its neighbors, and this feature can be captured by forming a graph neural network through message transfer, information propagation, and graph convolution.

For a given undirected graph G, each of its nodes $x_{i}$ has its own feature, and the edge connects two nodes $x_{i}$ and $x_{j}$ is $x_{ij}$, and the graph-aware hidden state of $x_{i}$ nodes at time t is $h_{i}^{t}$. $adj(x_{\left(i\right)})$ represents the characteristics of the edge, $adj(x_{i})$ represents the characteristics of adjacent points, $adj(h_{i}^{t})$ represents the state of adjacent nodes at time t, and f represents the parameter; the i node state at the next moment is shown in Formula (10).

(10) $$h_{i}^{t+1}=f(x_{i},adj(x_{\left(i\right)}),adj(h_{i}^{t}),adj(x_{i}))$$

For graph G(V,E), V is the set of nodes in the graph, E is the set of edges, the adjacency matrix A is used to represent the connection relationship between the nodes, and the degree matrix D is the number of connected nodes of each node, so the adjacency matrix A is normalized as shown in Formula (11).

(11) $$A=D^{-\frac{1}{2}}(A+I)D^{-\frac{1}{2}}$$

For the graph neural network, $H^{i}$ represents the node features of layer i, and $W^{i}$ represents the weight. The layer transfer relationship is shown in Formula (12).

(12) $$H^{i+1}=\sigma \left(AH^{i}W^{i}\right)$$

#### 3.2.2. DCGNN Architecture

The DCGNN model mainly includes the following modules:

Graph learning layer: a hidden graph adjacency matrix for learning the graph convolutional modules, which describes the connections between the nodes in the graph.

Graph convolution module: used for the convolution operation on the graph structure to capture the dependence in space.

Temporal convolution module: used for convolution operation on time series data to capture temporal dependence. Specifically, the receptive field size of the temporal convolution module can be adjusted by controlling the hyperparameters of the inflation factor.

Output module: the final output used to generate the model.

Modules are associated using both residual connection and jump connection. Residual connection and jump connection are two commonly used ways of achieving neural network connection; in residual connection, the input of the network is then directly added to the output of the network. Specifically, in a layer of the neural network, if the input is *x* and the output is H(X), the output of the residual connection is x+H(x). This is designed to allow the network to learn the residuals (i.e., the difference between H(X) and (x) rather than directly learning the complete mapping. Residual connectivity allows the gradient to propagate more easily to shallow layers, thus alleviating the problem of the gradient vanishing and accelerating the training of the network. The jump connection connects the input of a certain layer directly to the output of the subsequent layers rather than adding the output of the subsequent layers as in the residual connection. Jump connections allow information to spread deeper into the network faster, improving the training speed and model performance of the network.

**Figure 2:**
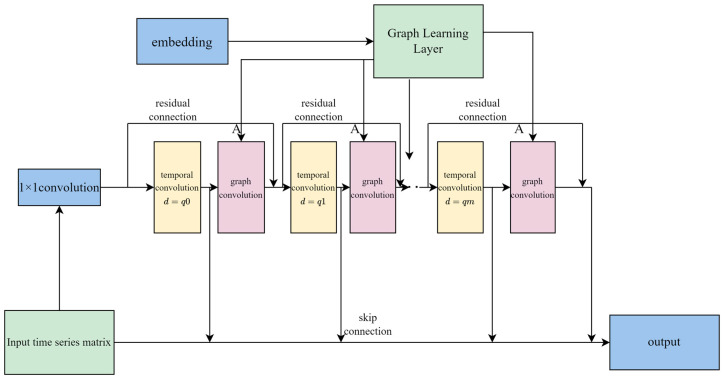
Structure diagram of the DCGNN algorithm.

As shown in Figure 2, first, 1 × 1 convolution projects the input into the hidden space. Second, temporal and graph convolution modules interleave to capture temporal and spatial dependence, respectively. The hyperparameter controlling the size of the receptive field of a temporal convolutional module, the inflation factor d, increases at an exponential rate of q. The inflation factor determines the distance between the neighboring elements in the convolution kernel. If the inflation factor is set to 1, the expansion convolution is equivalent to an ordinary convolution operation. However, if the inflation factor is set to an integer value greater than 1, the interval between the elements in the convolution kernel will increase accordingly so that the convolution operation can skip a certain number of input data points for calculation, thus expanding the receptive field of the convolution kernel on the input data. The graph learning layer is used to learn the hidden graph adjacency matrix of the graph convolutional modules. Resiresidual and hopping connections were added to the model to avoid the problem of a vanishing gradient. The DCGNN model graph is shown in Figure 2, where m is the number of graph convolutional modules and temporal convolution modules.

The graph learning layer captures the hidden relationship between variables from the time series by adaptively generating an adjacency matrix. Assuming that the relationship between the nodes in the graph is unidirectional, the change in one node feature can lead to a change in the other nodes, as shown in Formulas (13) to (18).

(13) $$M_{1}=tanh(\alpha E_{1}\theta_{1})$$

(14) $$M_{2}=tanh(\alpha E_{2}\theta_{2})$$

(15) $$A=ReLU(tanh(a(M_{1}M_{2}^{T}-M_{2}M_{1}^{T})))$$

(16) for i=1,2…N

(17) idx=argtopk(A[i,:])

(18) A[i,−idx]=0

where $E_{1}$, $E_{2}$ represent the randomly initialized node embeddings, which are learnable during training. $\theta_{1}$, $\theta_{2}$ are the model parameters, and α is the hyperparameter controlling the saturation rate of the activation function. arptopk(A[i,:]) is the index of the first k maxima of the return vector. The asymmetric character of the graph adjacency matrix is realized through Formula (15). Where the subtraction term and the *Relu* activation function regularize the adjacency matrix. Formulas (17) and (18) present a strategy that sparse the adjacency matrix while reducing the computational cost of subsequent graph convolution. For each node, we choose its first *k* closest nodes as its neighbors. While retaining the weights of the connected nodes, we set the weights of the unconnected nodes to zero.

**Figure 3:**
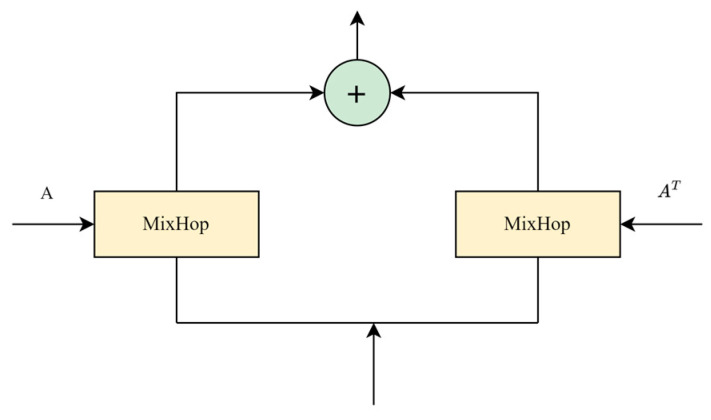
Structure diagram of graph convolution module.

Figure 3 shows the structure of the graph convolutional module and the MixHop propagation layer. Given the graph adjacency matrix, we propose using the MixHop propagation layer to handle information flow on spatially related nodes. The MixHop layer is a variant of a graph convolutional layer that includes information propagation steps and information selection steps to better capture the local structure between nodes when processing the graph data. In the information propagation step, the MixHop layer first disseminates the information horizontally by integrating the features of the nodes with those of their neighbor nodes. The purpose of this step is to enable each node to obtain information about its neighbor node. Typically, aggregation operations can use the form of feature-weighted sums of neighbor nodes, and the weights can be fixed or obtained by learning. In the information selection step, the MixHop layer vertically selects the information; that is, the multi-layer perceptron (MLP) is used to process and select the information obtained via horizontal transmission. The goal of this step is to allow the nodes to selectively integrate that information that is useful for the current task, thereby improving the representational power of node representation.

The graph convolution module is used to integrate the information of the nodes and adjacent nodes and contains two MixHop layers, which process the input and output information of the individual nodes. The structure diagram of the graph convolutional module is shown in Figure 3.

**Figure 4:**
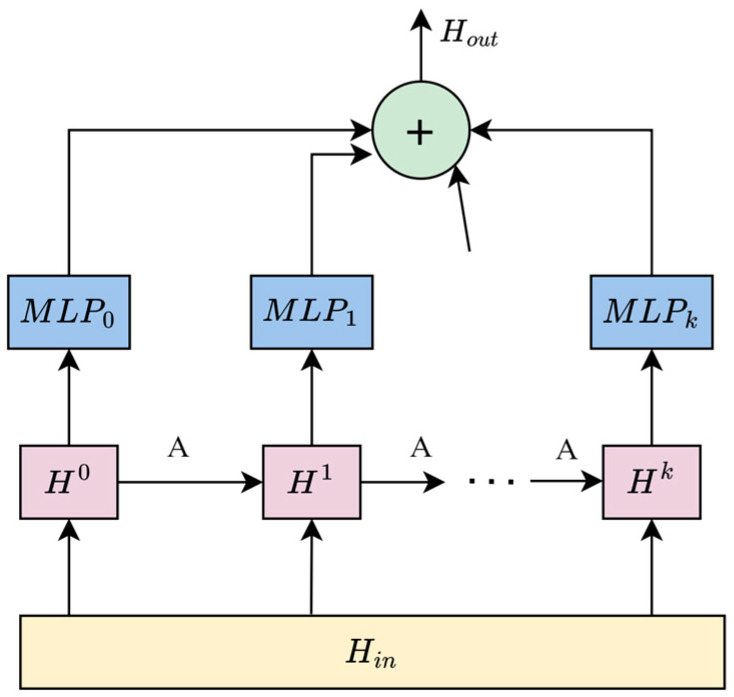
Structure diagram of MixHop layer.

The MixHop layer contains the information propagation and selection steps. It first propagates the information horizontally and then selects it vertically. The MLP represents a multi-layer perceptron. A structure diagram of the MixHop layer is shown in Figure 4.

Through adding an initial node information preserving factor excessive smoothing is alleviated, as shown in Formula (19).

(19) $$H^{\left(k\right)}=\beta H_{in}+\left(1-\beta \right)\tilde{A}H^{\left(k-1\right)}$$

where β is the hyperparameter used to maintain a proportion of the original node information. $H_{in}$ represents the output hidden state of the previous layer, $H_{in}=H^{\left(0\right)}$, $H_{out}$ represents the output of the current layer, and *k* indicates the depth of the propagation layer. $\tilde{A}={\tilde{D}}^{-1}(A+I)$, and ${\tilde{D}}_{ij}=1+\sum_{j}A_{ij}$.

To avoid information loss during the calculation, a parameter matrix is introduced as a feature selector, as shown in Formula (20).

(20) $$H_{out}=\sum_{i=0}^{k}H^{\left(k\right)}W^{k}$$

where $W^{k}$ represents the parameter matrix.

**Figure 5:**
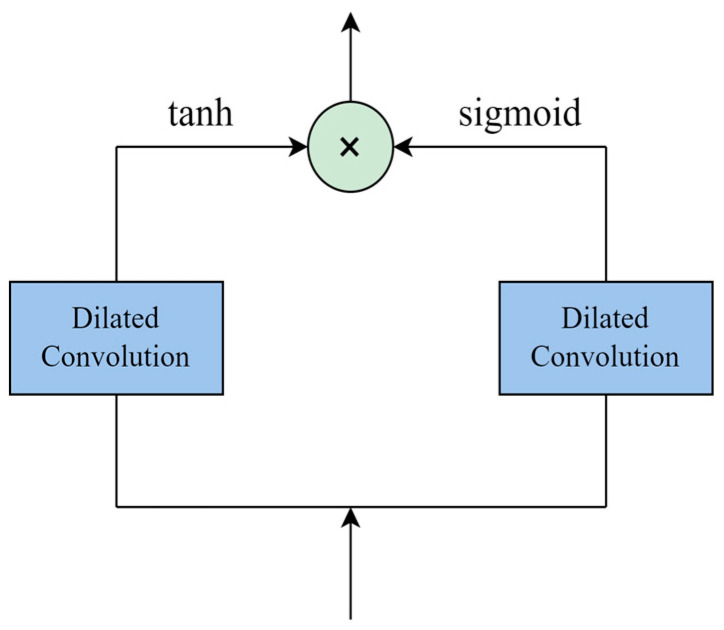
Structure diagram of temporal convolution algorithm.

Figure 5 shows the structural diagram of the dilated convolution layer. The temporal convolution model uses a one-dimensional dilated convolution filter to extract the temporal features. The temporal convolution model consists of two dilated convolution layers; one of the dilated convolution layers is followed by a tanh activation function that acts as a filter; the other dilated convolution layer is followed by an s-type activation function as a gate to control the filter and transfer the information to the next module.

Since the state variable graph is an undirected band-weight graph, the element $d_{ii}$ in its degree matrix D’ is represented in Formula (21).

(21) $$d_{ii}=\sum_{j=1}^{n}A_{ij}^{’}$$

Its symmetric normalized adjacency matrix $A^{’}$ is calculated as shown in Formula (22).

(22) $$A’={D^{’}}^{-\frac{1}{2}}(A’+I)D{’}^{-\frac{1}{2}}$$

To reduce computational complexity in MTGNN, the normalized weighted graph adjacency matrix, which is learned by the graph learning layer and is asymmetric, is made asymmetric by multiplying it with the upper triangular matrix, U. The formula is provided in Formula (23).

(23) A’=A’⊙U

Because the high similarity of the DCGNN algorithm is artificially set, it has half the number of input nodes. In this study, setting a supervised matrix *A*’, we calculate the distance between the adjacent matrix information obtained by Formulas (13)–(15) and the distance between *A*’, keeping the minimum distance between the two, and taking it as the final learning result.

The *F*-norm of the matrix difference is used to measure the distance dist(A) between the adjacency matrix and the supervised matrix learned by the graph learning layer, as shown in Formula (24).

(24) $$dist(A)={\left\|A^{’}-A\right\|}_{F}={\left(\sum_{i=1}^{m}\sum_{j=1}^{n}{\left(a_{i,j}^{’}-a_{i,j}\right)}^{2}\right)}^{1/2}$$

By manually designing the adjacent matrix of the retention graph learning layer, the unreasonable learning results are excluded to enhance the model’s ability to extract the characteristics of the hydropower unit data and to improve the prediction effect. Therefore, the graph learning layers of MFS-DSGNN are (13), (14), (25), (26), and (27).

(25) $$A=Relu(tanh(\frac{MinDist(A)}{dist(A)}a(M_{1}{M_{2}}^{T}-M_{2}{M_{1}}^{T})))$$

(26) for i=1,2,…,N j=1,2,…,N

(27) $$A\left[i,j\right]=A^{’}\left[i,j\right]=0\;A\left[i,j\right]<A^{’}\left[i,j\right],\;A\left[i,j\right]=A_{min}$$

where a is the hyperparameter controlling the saturation rate of the activation function, $A_{min}$ is the minimum value of matrix A, Dist(A) represents the set of the adjacency matrix and supervised matrix distances learned by the graph learning layer, and *MinDist* (*A*) represents the minimum value of all the distances currently calculated. With (13), (14), and (25), the adaptive establishment of the input sequence of the above figure can be achieved. Formulas (26) and (27) can eliminate the relationships that are not learned in the expected direction in order to realize the selection of the relationships and the sparse matrix.

## 4. Experiment

This paper takes a hydropower unit in Ertan Hydropower Station as the research object. Ertan Hydropower Station is a large-scale power station located in the southwest region of China equipped with six 550 MW Kaplan hydropower units (Zhangjiajie Tiancheng Electromechanical Equipment Manufacturing Co., Ltd., Zhangjiajie, China). The S8000 online monitoringsystem (Zhejiang Zhongzi Qing’an New Energy Technology Co., Ltd., Hangzhou, China) is used to monitor the operation states of the units [40]. The swing signal of the hydropower unit can be obtained through the S800 system installed in the power station. The swing signal mainly includes the displacement information of key positions, such as the upper guide bearing, lower guide bearing, and guide bearing around the main shaft; thus, it is an important basis for understanding the operating status of the various components inside the sets. It has a strong ability to characterize faults, damages, or abnormal situations.

The operating system used for this experiment is Windows 10. The Central Processing Unit (CPU) is an Intel(R) Core(TM) i9-9820X CPU@3.30GHz, and the Graphics Processing Unit (GPU) is an NVIDIA GeForce RTX 2080 Ti. The deep learning framework utilized is Tensorflow.

**Figure 6:**
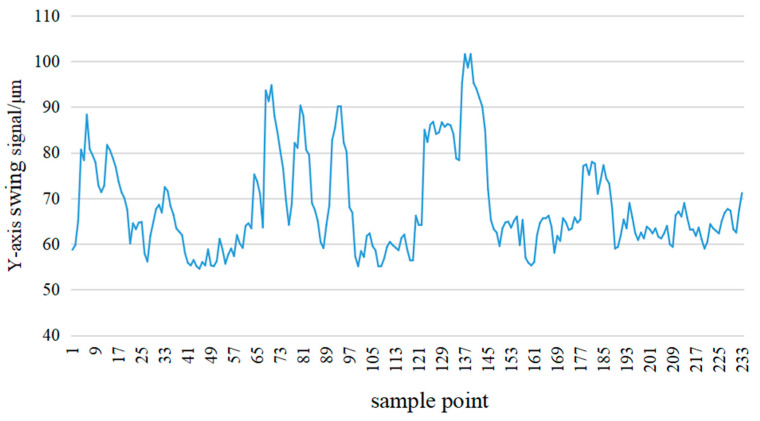
*Y*-axis swing signal of main guide bearing.

We selected the *Y*-axis swing signal of the guide bearing from 5 April to 5 October 2022 for training and testing, and the sampling interval was 1 min. Here, 70% of the total data are used as training samples, while the remaining 30% are test samples. Figure 6 shows the monitoring data of the *Y*-axis swing signal of the guide bearing from 6 to 8 July 2022.

**Figure 7:**
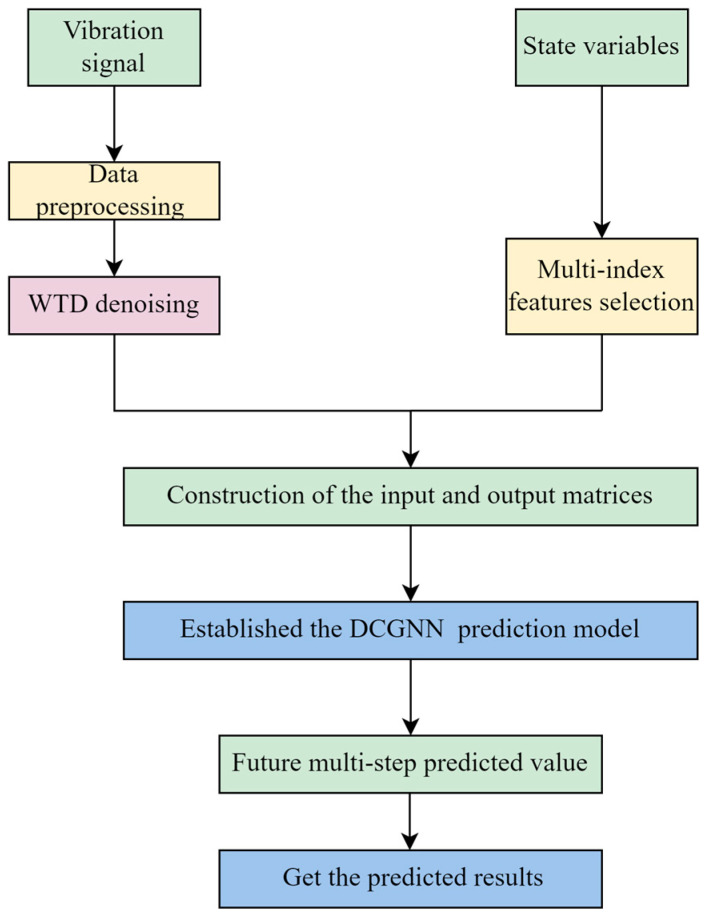
Flow chart of the MFS-DCGNN prediction model.

Research on the prediction of the swing trend of main guide bearings based on MFS-DCGNN includes two parts: model establishment and model prediction. The flow chart of the MFS-DCGNN prediction model is shown in Figure 7.

Model establishment: First, obtain the state variables $A=\{a_{1},a_{2},\ldots ,a_{n}\}$ and swing sets $B=\{b_{1},b_{2},\ldots ,b_{t}\}$, where *n* is the number of variables, and *t* is the sequence length. Second, the above swing signal *B* is denoised and filtered based on WTD (wavelet threshold denoising); then, the fusion feature index CF(X,Y) is used to select the features with strong correlation, forming the optimal feature subset $S=\{s_{1},\ldots ,s_{i},\ldots ,s_{p}\}$, standardize the denoised swing signal with the feature subset, and construct the input and output sample matrix. To perform this, first obtain the real-time state variable set and vibration set; standardize the above signal by Z-score, and build the input matrix; namely, the standardized signal is organized by time step, and each row represents the feature value of a time point. Therefore, the input matrix will consist of normalized signals with the number of rows as the length of the time series data and the number of columns as the number of features. The output matrix contains the normalized subset of features as the value of the target variable. Each row represents a sample (i.e., a time point), and each column represents a feature. Finally, the constructed input matrix is input into the trained MFS-DCGNN prediction model in the offline stage to obtain the state trend of the hydropower unit.

Model prediction: The real-time state variable set $A^{’}$ and the swing set were acquired, the Z-score was normalized to the above signals, and the input matrix was constructed. The constructed input matrix was inserted into the trained MFS-DCGNN prediction model to obtain the prediction results of the swing trend of the main guide bearing.

In order to evaluate the effectiveness of the proposed MFS-DCGNN prediction algorithm, the root mean square error (RMSE), mean absolute error (MAE), and correlation coefficient (CORR) were selected as the evaluation indicators. Among them, RMSE was used to measure the stability of the deviation degree between the predicted value and the true value, and MAE was used to reflect the average absolute deviation degree between the predicted value and the true value. CORR was used to reflect the degree of the linear relationship between the predicted value and the true value. The calculation formula is as follows:

(28) $$RMSE=\sqrt{\frac{1}{N}\sum_{i=1}^{n}{\left(H_{i}-T_{i}\right)}^{2}}$$

(29) $$MAE=\frac{1}{N}\sum_{i=1}^{n}\left|H_{i}-T_{i}\right|$$

(30) $$CORR=\frac{Cov(H,T)}{\sigma_{H}\sigma_{T}}$$

where H represents the predicted value, T represents the true value, and N represents the sequence length. $\sigma_{H}$ represents the standard deviation of the true values, and $\sigma_{T}$ represents the standard deviation of the predicted values.

Since the learning performance of this model is affected by the relevant parameter configuration of the model, appropriate parameters need to be selected to obtain better prediction results. The experimental parameters involved in this model mainly include the following: the size of the adjacency matrix, training times, training batch size, dropout rate, loss function, GCN layer depth, coefficient of dilated convolution, number of residual connection channels, number of jump connection channels, node dimension characteristics, weight attenuation, learning rate, etc.

The size of the adjacency matrix is the number of selected attributes. The number of training iterations is set to 50, with a training batch size of 32. The dropout rate is set to 0.3. The loss function is chosen as L1Loss. The GCN depth is set to 2. The dilated coefficient for dilated convolution is 2. The number of channels for residual connections is 8, and for skip connections, it is 16. The node input feature dimension is 24 × 7. Weight decay is set to 0.001, and the learning rate is set to 0.001.

### 4.1. Data Preprocessing

Before time-series prediction, the WTD algorithm is used to denoise and filter the original swing signal in order to effectively distinguish the high-frequency swing signal and noise and retain the real information. The wavelet basis function and the number of WTD algorithms have a direct influence on the signal-denoising effect. For different signals, the appropriate wavelet basis function can cause the noise to be clearly reflected in the wavelet coefficient, making the noise easy to filter out, achieving a better noise cancellation effect. The number of wavelet decomposition layers determines the degree of denoising. An excessive number of decomposition layers will lead to the loss of important signal information and increase the number of calculations; an inadequate number of decomposition layers will ineffectively filter the noise.

The denoising effect of the hydropower unit’s vibration signal directly affects the accuracy of trend prediction. In this study, WTD is used to filter the vibration signal. The purpose of WTD is to separate the signal from the noise by setting an appropriate threshold of φ; if the wavelet coefficient is greater than this threshold, we assume that it is caused by the signal and, if it is less than this threshold, we assume that it is caused by the noise. After wavelet transformation, the real signal energy is concentrated on several wavelet coefficients with large amplitudes, while the noise energy is concentrated on all the wavelet coefficients. Accordingly, the noise can be eliminated by reducing the wavelet coefficient caused by the noise.

Assuming that the original swing signal can be described as f(t)=s(t)+n(t), where s(t) is the real swing signal and n(t) is the Gaussian white noise following the $N(0,\sigma^{2})$ distribution; the wavelet threshold denoising steps are as follows:

(1) Decompose the original swing signal f(t), determine the wavelet base and wavelet decomposition level N, and obtain the wavelet coefficient φ;
(2) The 1 − *N* high-frequency coefficient after wavelet decomposition is the quantified threshold; a different threshold value is selected for each wavelet coefficient, and the wavelet coefficient of the noise is set to 0;
(3) The signal reconstruction is conducted to obtain the signal after denoising.

The key aspect of WTD is the selection of the threshold after the decomposition of the wavelet coefficient. If the threshold is set too high, the signal will be distorted, and the noise threshold will be preserved. The threshold is usually divided into a hard threshold (continuous data function) and a soft threshold (fixed value). As the hard threshold of the boundary discontinuity point shrinks until it reaches 0, it can control discontinuity. The signal does not easily produce deviations, and the fidelity of the reconstruction signal is high; thus, this study used the hard threshold with the minimum threshold method to determine threshold φ. Its essence is related to the fact that the original noise signal is similar to the unknown regression function; thus, the estimate in the given function achieves a minimized maximum mean square error. To estimate the threshold, the calculation formula is as follows:

(31) $$\varphi =\left\{\begin{array}{c} 0.3936+0.1829(\frac{lnN}{ln2}),N>32\\ 0,N\leq 32 \end{array}\right.$$

The denoising coefficient can be used as an indicator to measure the noise reduction effect, and its formula is expressed as follows:

(32) $$\lambda =10\log_{10}\frac{\frac{1}{n}\sum_{n}{\tilde{f}}^{2}(i)}{\frac{1}{n}\sum_{n}{\left[f(i)-\tilde{f}(i)\right]}^{2}}$$

where f(i) is the original signal, $\tilde{f}(i)$ is the signal after noise reduction, and n is the signal length. The smaller the *λ* value is, the better the noise reduction effect.

**Figure 8:**
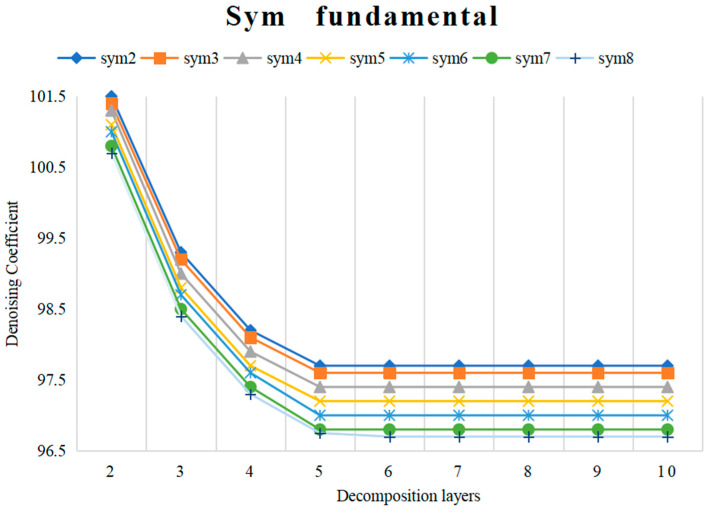
Denoising coefficient of Sym wavelet bases under different decomposition layers.

**Figure 9:**
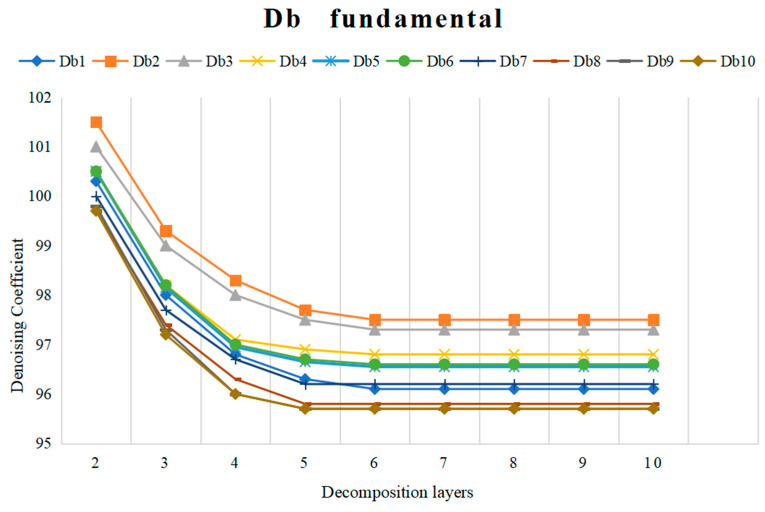
Denoising coefficient of Db wavelet bases under different decomposition layers.

In this study, we compare the denoising effects of the wavelet groups known as Symlets (Sym) and Daubechies (Db), including 17 wavelet groups: sym 2, sym 3, sym 4, sym 5, sym 6, sym 7, and sym 8 and Db 1, Db 2, Db 3, Db 4, Db 5, Db 6, Db 7, Db 8, Db 9, and Db 10. Figure 8 and Figure 9 show the denoising coefficients of the wavelet bases of Sym and Db at different numbers of decomposition layers. As can be seen in Figure 8, sym 8 has a low denoising coefficient and is the best. In Figure 9, Db 10 has a small denoising coefficient, and its value is less than that of sym 8. Furthermore, when the number of decomposition layers is five, the wavelet coefficient basically does not change.

**Figure 10:**
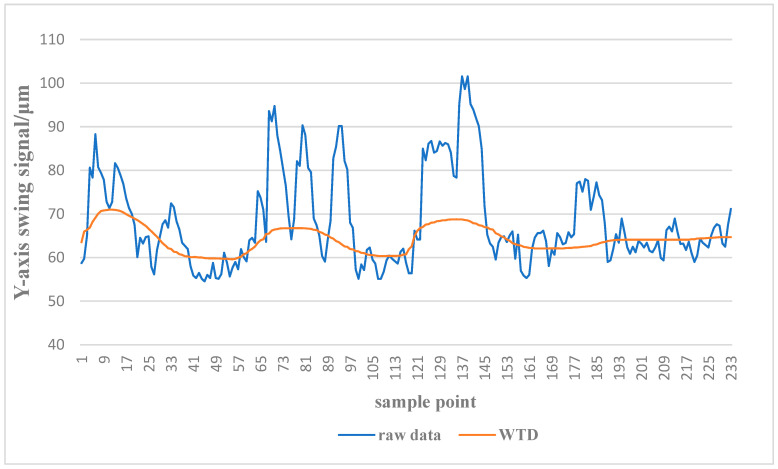
Raw data of the *Y*-axis swing signal and the data after the WTD.

Based on the experimental analysis presented above, Db 10 was selected as the optimal wavelet base of the WTD algorithm, and the number of decomposition layers was determined as five. Figure 10 shows the raw data of the *Y*-axis swing signal and the data after the WTD algorithm. The denoised data are smoother than the original data and preserve the running trend of the signal well. When the features are selected, the state variables associated with the *Y*-axis swing signal of the guide bearing can be excavated more accurately.

### 4.2. Results of MFS Algorithm

As the abnormal swing of the hydropower unit is mainly composed of various factors, such as electrical and mechanical factors and the hydraulic swing during operation, the influence of state variables should be considered when constructing the trend prediction algorithm. According to the manual experience analysis, the unit contains nine kinds of state variables: water head (H), active power (P), the temperature of the upper guide bearing oil groove (T), excitation current (I), excitation voltage (U), axial displacement (D), unit speed (S), pressure pulsation at the inlet of the guide vane and runner (V1), and pressure pulsation between the runner and the top cover (V2).

In order to select the state variables that best reflect unit operation, the multi-index feature selection algorithm is used to analyze the correlation between the state variables and the *Y*-axis swing signals from the linear and non-linear perspectives. Furthermore, the Pearson correlation coefficient can be sensitive enough to mine the linear relationship state variables of the *Y*-axis swing signal, while the distance coefficient mines the non-linear relationship and obtains the feature selection subset using the MFS algorithm.

**Figure 11:**
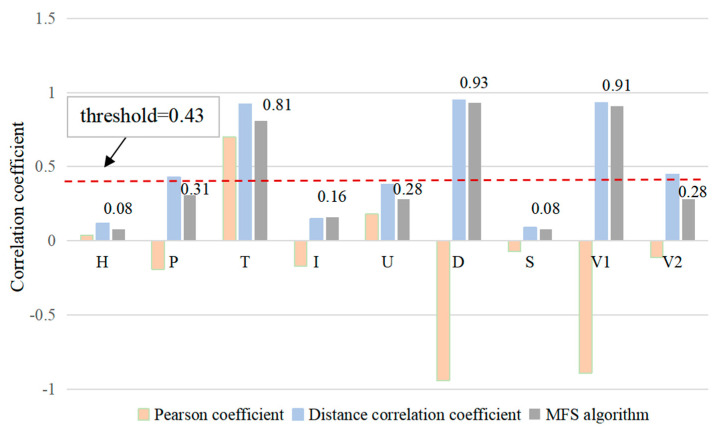
Comparative results of the correlation coefficient for 9 state variables.

Figure 11 shows the comparative results of the correlation coefficients from three algorithms (the Pearson correlation coefficient, distance correlation coefficient, and MFS algorithms) for nine state variables. Figure 9 shows that the mean correlation coefficient calculated by the MFS algorithm is 0.43. Therefore, 0.43 is set as the correlation threshold of the MFS algorithm. Among the coefficients, the MFS algorithm correlation coefficients of the upper guide bearing oil groove (T), axial displacement (D), and pressure pulsation at the inlet of the guide vane and runner (V1) are 0.81, 0.93, and 0.91, respectively; these values are greater than the correlation threshold of 0.43 and show a high correlation with the *Y*-axis swing signal. Therefore, the state variables of the upper guide bearing oil groove (T), axial displacement (D), and pressure pulsation at the inlet of the guide vane and runner (V1) are selected as the input of the prediction algorithm.

**Figure 12:**
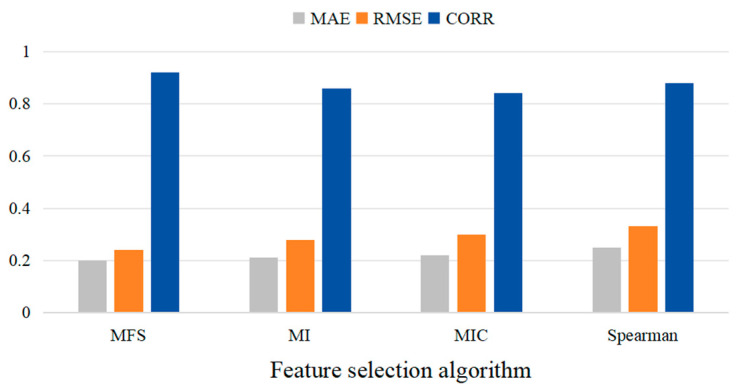
Comparative results of four different feature selection algorithms.

Figure 12 shows the comparative results of the MAE, RMSE, and CORR values of the four different feature selection algorithms. It can be seen that compared with the MI, MIC, and Spearman algorithms, the MAE of the MFS algorithm is decreased by 9.52%, 13.63%, and 20.83%, respectively; the RMSE is decreased by 6.89%, 12.90%, and 15.62%, respectively; and the CORR values are increased by 5.74%, 8.23%, and 10.84%, respectively.

### 4.3. Prediction Results of DCGNN Algorithm

**Figure 13:**
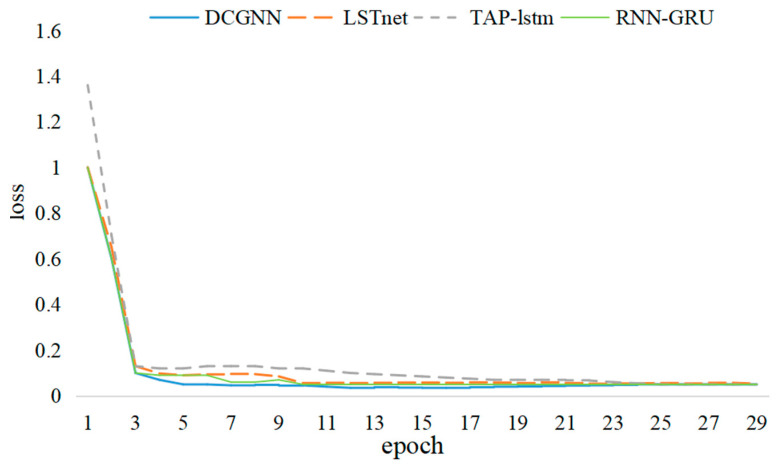
Loss values of DCGNN, LSTNet, TPA-LSTM, and RNN-GRU algorithms.

In this study, the DCGNN, LSTNet, TPA-LSTM, and RNN-GRU algorithms were trained on the *Y*-axis swing signal. Figure 13 shows that the DCGNN algorithm can converge faster than the other networks. At an amount equaling 30, the loss value of the above four algorithms fluctuates in a small range; therefore, the number of iterations is set to 30 in the comparisons.

In order to verify the effectiveness of the DCGNN algorithm, the dataset of the *Y*-axis swing signal of the hydropower units is used to compare the prediction results of the DCGNN algorithm, LSTNet algorithm, RNN-GRU algorithm, and TPA-LSTM algorithm.

**Figure 14:**
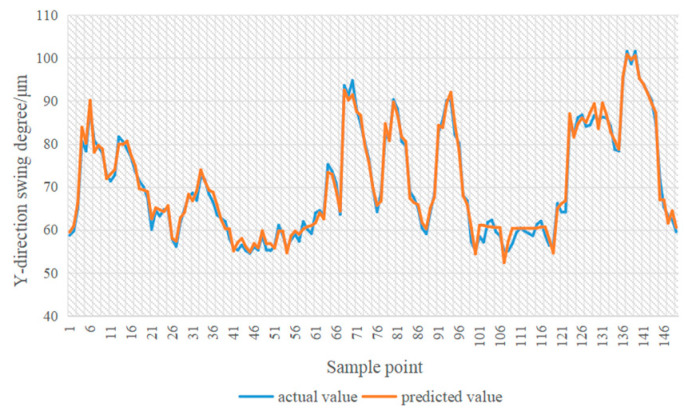
DCGNN algorithm.

**Figure 15:**
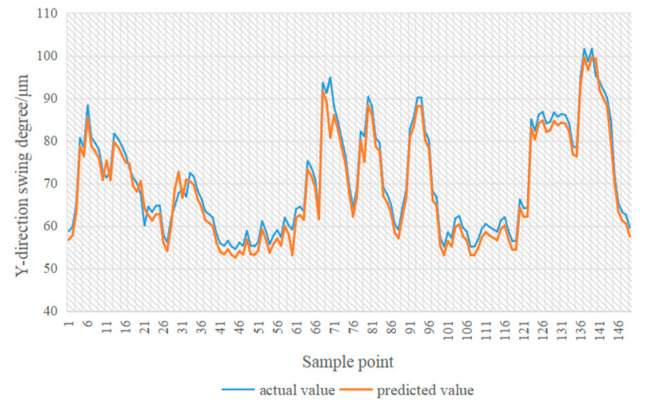
LSTNet algorithm.

**Figure 16:**
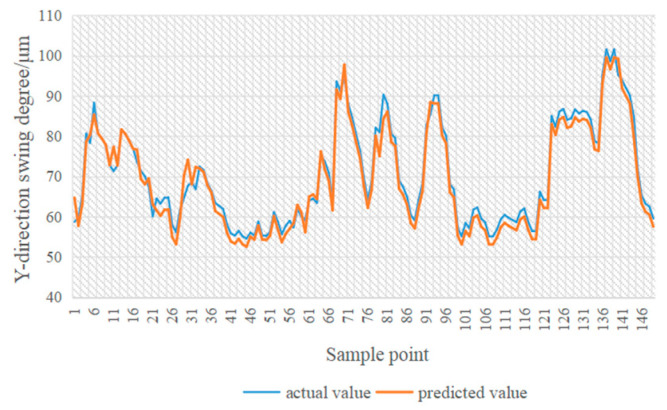
RNN-GRU algorithm.

**Figure 17:**
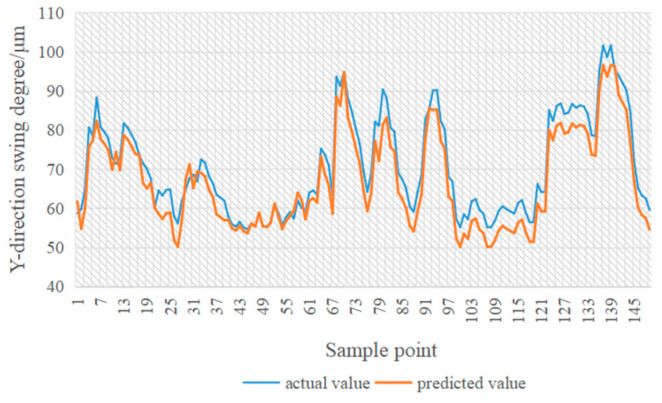
TPA-LSTM algorithm.

In Figure 14, Figure 15, Figure 16 and Figure 17, the blue line represents the actual value, and the red line represents the predicted value. The experimental results show that the TPA-LSTM algorithm has lower prediction accuracy. The RNN-GRU and LSTNet algorithms can fit the data trend, but the prediction results are affected by the length of the recurrent jump layer, which is set manually. The DCGNN algorithm predicts the results closest to the true values.

From Table 1, it can be seen that as the prediction steps increase, the prediction accuracy of each model gradually decreases. As the DCGNN algorithm considers the influence of other variables, the algorithm shows good prediction accuracy. At prediction step 3, the MAEs of the DCGNN algorithm are decreased by 6.05%, 6.32%, and 3.04%, respectively; the RMSEs are decreased by 9.21%, 9.01%, and 2.83%, respectively, compared with the RNN-GRU, LSTNet, and TAP-LSTM algorithms, and the CORR values are increased by 0.64%, 1.06%, and 0.37%, respectively.

**Figure 18:**
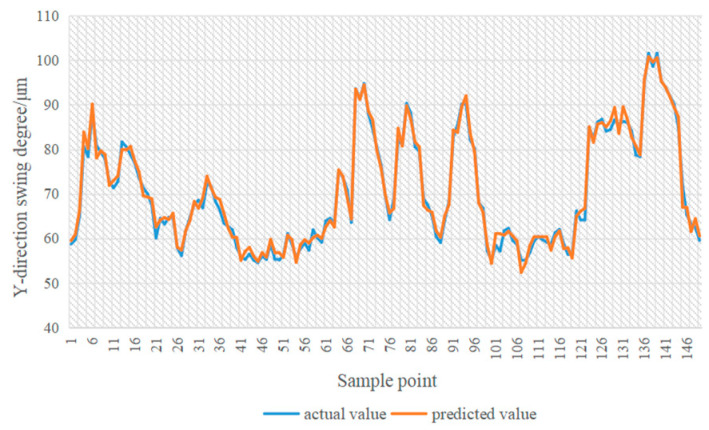
MFS-DCGNN algorithm.

**Figure 19:**
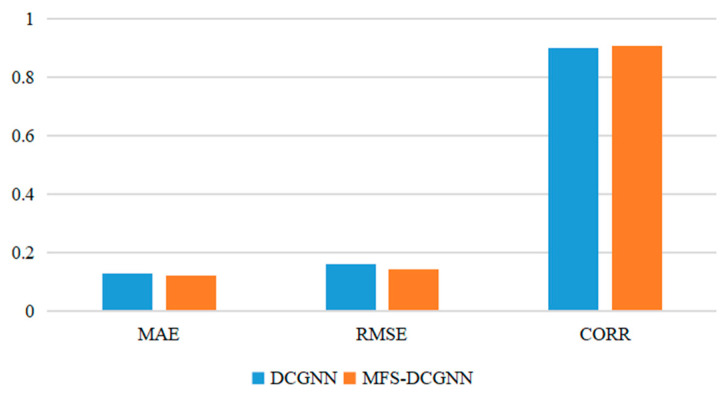
Comparative results of DCGNN and MFS-DCGNN algorithms.

According to Figure 18 and Figure 19, the MAE of the MFS-DCGNN algorithm is decreased by 7.70%; the RMSE is decreased by 11.88%; and the CORR value is increased by 0.69% compared with the DCGNN algorithm.

## 5. Conclusions

Swing trend prediction of a main guide bearing can obtain the future operation status, find operational abnormalities and faults as quickly as possible, avoid the occurrence of major accidents, and allow for a state maintenance plan to be made in a timely fashion, reducing economic losses. This paper proposed a prediction method for the swing trend of the guide bearing based on the MFS-DCGNN algorithm. First, this study considered the influence of various factors on the *Y*-axis swing signal of the guide bearing. Before swing trend prediction, the multi-index feature selection algorithm is used to obtain the appropriate state variable, and the low-dimensional effective feature subset is obtained through the Pearson correlation coefficient and distance correlation coefficient algorithms. Second, the DCGNN algorithm is used to predict the swing trend of the guide bearing. The existing GNN methods rely heavily on predefined graph structures for prediction. The DCGNN algorithm can solve the problem of spatial dependence between variables without defining the graph structure and can provide the adjacency matrix simulated by the graph learning layer, avoiding the problem of over-smoothing that often occurs in the graph convolution network and effectively improving the prediction accuracy. The experimental results showed that, compared with the RNN-GRU, LSTNet, and TAP-LSTM algorithms, the MAEs of the DCGNN algorithm were decreased by 6.05%, 6.32%, and 3.04%, respectively; the RMSEs were decreased by 9.21%, 9.01%, and 2.83%, respectively; and the CORR values were increased by 0.63%, 1.05%, and 0.37%, respectively. Compared with the DCGNN algorithm, the MAE of the MFS-DCGNN algorithm was decreased by 7.70%, the RMSE was decreased by 11.88%, and the CORR value was improved by 0.69%, and the prediction accuracy was effectively improved.

At present, hydropower stations are becoming increasingly intelligent, integrated, and complex, and the construction of an accurate health performance evaluation model is particularly important. With regard to the deep mining of state-monitoring data features, determining the most effective ways to use limited data to build high-precision and generalizable models is one of the research tasks currently being pursued. In practical engineering applications, the feature mining of monitoring data is also complex and challenging work, and different units and equipment types show great differences. In the era of big data, determining how to quickly and accurately mine the useful information needed by customers from such data and how to provide substantial guidance for the intelligent construction of hydropower stations is also a big problem. Despite the remarkable results of the method used in this study in current applications, we face a range of challenges in generalizing it to other categories of problems. First, parameter adjustment and optimization may need to be repeated for different application scenarios, increasing the complexity of implementation. Moreover, existing methods may encounter performance bottlenecks when handling large-scale or high-dimensional data, requiring further research and optimization to guarantee the efficiency and accuracy of the algorithm. Thus, while this approach demonstrates a broad potential for application, these challenges need to be thoroughly explored and overcome when extended to more categories of problems.

## Figures and Tables

**Table 1:** Prediction results of different algorithms with different prediction steps.

	Prediction Steps Algorithm	Evaluating Indicator		
		MAE	RMSE	CORR
3	DCGNN	0.1304	0.1616	0.90253
	RNN-GRU	0.1388	0.1780	0.8968
	LSTNet	0.1392	0.1776	0.8930
	TPA-LSTM	0.1345	0.1663	0.8992
12	DCGNN	0.1508	0.1965	0.8522
	RNN-GRU	0.1504	0.1901	0.8506
	LSTNet	0.1429	0.1927	0.8542
	TPA-LSTM	0.1535	0.1971	0.8493
36	DCGNN	0.1885	0.2360	0.7673
	RNN-GRU	0.1866	0.2345	0.7666
	LSTNet	0.1899	0.2352	0.7651
	TPA-LSTM	0.1911	0.2446	0.7494
72	DCGNN	0.2355	0.2973	0.6758
	RNN-GRU	0.2453	0.3017	0.6683
	LSTNet	0.2503	0.3871	0.6702
	TPA-LSTM	0.2489	0.3026	0.6647

## Data Availability

The data presented in this study are available on request from the corresponding author due to privacy restrictions.
